# Electroencephalogram Gamma Band Power Correlates with Anhedonia in a Community Sample

**DOI:** 10.3390/jpm15110536

**Published:** 2025-11-03

**Authors:** Sarah L. Coleman, Ian D. Evans, Christopher F. Sharpley, Vicki Bitsika, G. Lorenzo Odierna, Kirstan A. Vessey

**Affiliations:** Clinical Neuroscience Brain-Behaviour Research Group, School of Science and Technology, University of New England, Armidale, NSW 2351, Australia; scolem26@myune.edu.au (S.L.C.); ievans3@une.edu.au (I.D.E.); vicki.bitsika@une.edu.au (V.B.); godierna@une.edu.au (G.L.O.); kvessey@une.edu.au (K.A.V.)

**Keywords:** major depression, depressive subtypes, EEG, electroencephalography, anhedonia, anterior cingulate cortex

## Abstract

Major depression (MD) is a condition characterised by persistent sadness and apathy, sometimes accompanied by changes in sleep, appetite, and energy levels. It is highly heterogeneous, and depressive subtypes exhibit differing symptom profiles and patterns of brain activity. **Background/Objectives**: Currently, there are no physiological diagnostic means to detect depression or depressive subtypes. An emerging biomarker may be the electroencephalogram (EEG) band, gamma, due to the role of this frequency in reward processing and cognition. The aim of this work was to complete an exploratory study to investigate the interaction between gamma band power, depression, and four depressive subtypes. **Methods**: A correlative study between resting-state gamma band power and individual scores on the Zung Self-rating Depression Scale (SDS) was completed using exact standardised low-resolution electromagnetic tomography (eLORETA) using EEG data from a community sample of 100 participants, including not depressed and depressed participants, and four depressive subtypes (anhedonia-, cognitive- and somatic-depression and depressed mood). **Results**: There was no significant positive correlation between gamma band power and overall depression score. However, there was a significant positive correlation between anhedonia and gamma band power, predominantly in the left anterior cingulate cortex, which may be consistent with dysfunctional reward processing, a characteristic of anhedonia. Additional areas of significance included the posterior cingulate cortex and left middle and superior frontal cortex. **Conclusions**: These results provide preliminary support for neurophysiological indicators of depressive subtypes and may help inform diagnosis and treatment guidance for depression and depressive subtypes in the future.

## 1. Introduction

Major depression (MD), commonly known as depression, is characterised by persistent sadness and a loss of interest in activities, accompanied by emotional and cognitive changes in behaviour [1,2]. MD arises from complex biological, behavioural, psychosocial, and cultural interactions [3,4,5,6]. It is projected to become the greatest contributor to mental illness by 2030 [1,2]. Within and between individuals, depression can be a highly inconsistent condition, encompassing various combinations of core clinical features such as persistent sadness or low mood, loss of interest in activities once enjoyed, low energy, disrupted eating and sleeping patterns, feelings of worthlessness or guilt, difficulty concentrating, and in extreme cases, suicidal thoughts [7]. This means diagnosis and treatment can be challenging [8].

Objective diagnosis of depression, unaffected by clinical bias, may be aided by novel biomarkers [9,10]. Although identification of reliable biomarkers for depression has proven challenging [11,12], electroencephalography (EEG) has shown promise as a diagnostic marker and predictor of treatment response [2,13]. EEG detects rhythmic pulses in electrical activity of the brain, known as neural oscillations, which are defined according to their frequency: delta (up to 4 Hz), theta (4–8 Hz), alpha (8–12 Hz), beta (12–30 Hz), and gamma (30–200 Hz) [14]. MD patients display abnormal EEG activity under resting and task-related conditions in contrast to healthy controls; therefore, abnormal EEG signals may be considered as an objective measure for diagnosis, screening, or treatment response in depression [2,13,14,15]. Of the EEG waveforms, gamma rhythms have been identified as one of the more likely to be reliable EEG biomarkers of depression [16,17]. Gamma oscillations are associated with higher-order perception, reward processing, and cognition [18], which may be inhibited in MD, where two of the major diagnostic criteria are “diminished interest or pleasure” and “diminished ability to think or concentrate” [7]. Studies have suggested disruptions in gamma oscillations are a candidate biomarker for depression; however, the heterogeneity of findings, methodology, and gamma frequency range in previous EEG gamma band-MD research has delayed translation into clinical application [16,17].

Another difficulty in finding a unitary biomarker for depression is its heterogeneity. Depression has traditionally been treated as a single disorder; however, the behavioural symptoms of MD are broad [19], in which two people could receive a MD diagnosis yet share no symptoms [20]. To move beyond this issue, research has attempted to cluster and group depressive symptoms into subtypes of depression [21,22]. One method of subdividing depressive symptoms was developed by Sharpley and Bitsika, who proposed four depressive subtypes according to common clusters of symptoms and neurobiological and behavioural substrates: Depressed Mood, Anhedonia, Cognitive Depression, and Somatic Depression [22]. Depressed mood includes depressed mood for most of the day, unrealistic guilt or worthlessness, and thoughts of suicide. Individuals suffering from anhedonic depression experience withdrawal from reward-seeking behaviours. Cognitive depression is expressed as deficits in executive functioning, resulting in difficulty shifting mindsets, and is prone to negative bias. Somatic depression is characterised by an imbalance of the somatic symptoms associated with depression, such as appetite, sleep, and fatigue [22]. These subtypes demonstrated reliability and validity in different populations [23].

While previous research has shown that MD might correlate with high gamma band power [17], no study to date has carefully investigated EEG gamma power and these four subtypes of depression [22]. The purpose of this study was to investigate the possibility that resting-state EEG gamma power may be used for differentiating between individuals with depression, and also the four depressive subtypes (depressed mood, anhedonic depression, cognitive depression, and somatic depression) [22]. In order to evaluate this, the current study was focused on the general community rather than severely depressed individuals who might be unable to self-care, in order to maximise the generalizability of results to the wider population. Zung Self-rating Depression Scale (SDS) scores were correlated with EEG resting state, gamma power across brain regions using eLORETA, and significant associations with gamma power and overall MD, and the four subtypes of depression described above were evaluated.

## 2. Materials and Methods

### 2.1. Participants

A sample of 101 participants was drawn from a larger study investigating mental health as part of the New England Mental Health Study. One subject was excluded due to a lack of usable EEG data, resulting in a final sample size of 100 (44 males and 56 females). These participants were selected based on not having a medical history of severe physical brain injury, brain surgery, or history of epilepsy or seizure disorder. Participants read an explanatory statement and were allowed to ask questions before providing written consent to participate. Ethics approval for the study was provided by the Human Research Ethics Committee of the University of New England, Australia (Approval No. HE14-051, 25 March 2014), consistent with the Code of Ethics of the World Medical Association (Declaration of Helsinki).

### 2.2. Depression Scales

The Zung Self-rating Depression Scale (SDS) [24] consists of twenty items to assess depressive symptoms based on the most recent definition of major depressive disorder [7]. Ten items are worded positively and ten negatively. Respondents indicate the frequency with which they experienced the symptoms corresponding with the 20 SDS items during the past two weeks by scoring 1 (“none or a little of the time”), 2 (“some of the time”), 3 (“good part of the time”), or 4 (“most or all of the time”) providing raw scores of 20 to 80 [24,25]. A total score of 40 or above indicates “clinically significant depression” [25], and 33 members of the current sample of participants were in excess of this threshold. The SDS has split-half reliability of 0.81 [24], 0.79 [26], and 0.94 [27], and internal consistency (Cronbach alpha) of 0.88 for depressed patients and 0.93 for non-depressed patients [28]. Rather than classify “depressed” and “non-depressed” patients based on a SDS score ≥ 40, the total SDS and subtype scores of the four depressive subtypes, derived from the SDS as determined by Sharpley and Bitsika [23], were calculated for each individual and used in correlational analyses. This method enabled all symptom profiles from participants to be used in the data analysis. The allocation of SDS questions according to the four depressive subtypes and the brief profile is presented in Table 1. For more information on the four depressive subtypes of depressed mood, anhedonic depression, cognitive depression, and somatic depression, see Sharpley and Bitsika [22].

### 2.3. Electroencephalogram Data Processing

EEG data from participants were collected using a 40-channel Neuroscan QuikCap EEG machine (Compumedics USA 144 Ltd., El Paso, TX, USA). Electrodes composed of sintered Ag/AgCl were placed according to the 10–20 system of the International Federation and aligned with anatomical inion and nasion points. A NuAmps digital amplifier (Compumedics USA 144 Ltd., El Paso, TX, USA) acquired and digitised signals at a sampling rate of 1000 Hz and passed them through a bandpass filter of DC to 250 Hz. The amplifier was connected to Curry 7 Acquisition software (Dell, Compumedics USA 144 Ltd., El Paso, TX, USA) running on a Dell Optiflex 9020 desktop PC (Dell Inc., TX, USA). Recordings were referenced to the average of the A1-A2 earlobe electrodes and later converted to a common average reference offline. Electrooculogram (EOG) data were collected using four electrodes with two arranged above and below the left eye, measuring vertical eye movement, and two more arranged outside the left and right canthi, measuring horizontal eye movement. EOG is recorded concurrently with EEG recording to find coefficients for sources of noise such as blinking or eye movement artefacts [29]. Prior to the start of recording, impedance values at all electrodes were <10 kΩ, ensuring the quality of signal acquisition.

During EEG acquisition, participants were seated in an experimental booth with headphones placed over the ears, minimising external stimuli, and were asked to relax. After a 15 min adaptation period, continuous EEG data were collected for 3 min eyes open and 3 min eyes closed. At the end of the protocol, the experimental equipment was removed, and participants were thanked for their involvement.

EEG signal processing was previously described [30]. Briefly, signals were filtered using a 1–45 Hz 2nd-order Butterworth bandpass filter, then referenced to the common average (as identified above). A Hann window with 10% width to prevent data loss was used to filter data. The data were visually examined to identify artefacts, including eye movements, muscle movement, spontaneous discharges, or electrode pops, which were removed. Bad block and eye blink detection, using the magnitude of eye blink deflections as a set threshold criterion to detect artefacts, was undertaken by three automated methods (Subtraction, Covariance, and Principal Component Analysis) to produce clean EEG data.

From the cleaned EEG data, back-to-back 2 s epochs were created. Any epochs with bad blocks were excluded from the averaged data. The majority of participants had over 90% usable and artefact-free epochs for both eyes open and eyes closed conditions, with the lowest frequencies of usable epochs being 87% in the eyes open condition and 49% in the eyes closed condition.

The 2 s EEG epochs for each participant for eyes open and closed were averaged separately to calculate the cross-spectra in gamma band (30–45 Hz) for both resting-state conditions. Gamma band frequency was capped at 45 Hz to reduce noise from physiological (e.g., electromyogram, electrooculogram) and non-physiological artefacts (e.g., power line noise, electrical devices) [31]. Each cross-spectra file was transformed into a corresponding file using the Key Institute LORETA (low-resolution brain electromagnetic tomography) [32]. This software computes images of electrical neural activity from EEG and processes them into a 3D distribution of activity in the brain. Transformation of cross-spectra files, computations, and analysis using exact low-resolution brain electromagnetic tomography (eLORETA) software was conducted. Computations in eLORETA (version 20240713) were made in a standardised, realistic head model [33] based on the MNI (Montreal Neurological Institute) template [34].

Statistical non-parametric mapping (SnPM) of eLORETA data was used to correlate gamma band power in eyes closed and eyes open resting conditions with each of the depression scores (total depression, depressed mood, anhedonia, cognitive depression, and somatic depression). This analysis was performed ten times (eyes open or eyes closed), producing resting-state gamma power for each depression score. SnPM of eLORETA single group regression analysis was performed each time with all voxels, for all time samples and discrete frequencies, and with no data normalisation [35]. SnPM of the eLORETA images was performed for each correlation using the built-in voxel-wise randomisation tests (5000 permutations) and employed a log-F-statistic for dependent groups with corrected critical thresholds and *p*-values [35]. Voxels with positive or negative correlations were considered significantly different between non-depressed and depression/subgroups when (*p* < 0.05) [36]. From this, the significant voxel, Brodmann areas, and MNI-brain coordinates were noted. All demographic data and psychological variables (i.e., SDS scores) were analysed using SPSS version 29 (IBM Corp., Armonk, NY, USA).

## 3. Results

### 3.1. Age, Sex, and SDS Scores

The age, gender distribution, and average total SDS scores for this cohort are provided in Table 2. Mean age of the group was 32.53 (SD 14.13; range 18–75 years). The internal consistency (Cronbach’s alpha) for SDS was 0.921, indicating satisfactory reliability. Normal Q-Q plots for the SDS revealed close to a straight line, reflective of normality. The mean SDS score was 36.70 (SD = 11.25), minimum = 21, and maximum = 66. According to Zung’s [25] cutoff score of 40, 33 participants had clinically significant depression (mean SDS score 50.39, SD 7.43) and 67 participants did not (mean SDS score = 29.95, SD = 4.83; *F* (1,99) = 273.729, *p* < 0.001, ƞ_p_^2^ = 0.736). No significant correlations were found between age or sex and SDS total score or any of the four SDS subtype scores, as shown in Table 3. However, significant positive correlations between total SDS score and the four subtypes of depression were observed (Table 3).

### 3.2. Depressive Subtypes

Mean scores for the four depressive subtypes using the SDS are tabulated in Table 4. Although Table 4 displays the mean of the SDS actual scores, to allow for depressive subtype weighting to be equal (SDS items are not evenly distributed across depressive subtypes; see Table 1 for additional detail), scores were averaged before correlational analysis. All four SDS subtype scores significantly correlated with each other (all ρ ≥ 0.626, *p* < 0.001), as seen in Table 3.

### 3.3. Gamma Band

**Figure 1:**
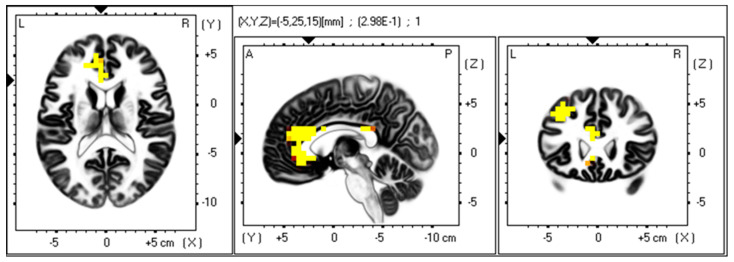
Voxel map for regions of significant gamma band power associated with the anhedonic depression subtype. eLORETA was used to generate a voxel-wise, statistical nonparametric map of all participants to correlate EEG gamma band power (30–45 Hz) in the eyes open resting state with anhedonia scores. Voxels with a 0.05 significance level are indicated as a heatmap (yellow/orange/red) after correction for multiple comparisons in the horizontal (**left**), sagittal (**middle**), and coronal (**right**). Structural anatomy is shown in greyscale. The axial, sagittal, and coronal planes show the same activation area. Abbreviations: L, left; R, right; A, anterior; P, posterior.

Using exact low-resolution electromagnetic tomography (eLORETA), 3D voxel-wise statistical nonparametric maps for gamma band power and SDS scores were computed to show cortical areas that exceeded the significance threshold (*p* < 0.05) and were marked in yellow. This showed which brain regions demonstrated the most meaningful association with the gamma band and SDS scores. A representative analysis for the anhedonic depressive subtype in the eyes-open resting-state is presented in Figure 1. The brain area demonstrating the highest activation was in the anterior cingulate cortex (Figure 1). Other structures demonstrating significant positive correlations include the left posterior cingulate and left middle and superior frontal gyrus (Figure 1).

Correlation analyses were performed in eLORETA using each depression score (major depression and the four subtypes) and gamma band power. Results for the brain regions with the highest correlation with the depression score are shown in Table 5 for eyes-open resting-state. For the eyes open condition, in major depression and the depression subtypes, the left anterior cingulate cortex was the brain region with the highest EEG gamma band power correlation with depression, regardless of subtype (Table 5). However, the only significant positive correlation was found between gamma band power and the anhedonia subtype (*r* = 0.298, *p* = 0.032, two-tailed), with no other correlations reaching significance. For the eyes closed condition, there were no significant correlations between major depression, depression subtypes, and the EEG gamma band power (Table 5). Additionally, there was no clear consistency in the brain regions identified between depression subtypes and the EEG gamma band power for the eyes closed resting-state condition (Table 6).

## 4. Discussion

This study employed eLORETA brain region mapping to test for the presence of significant correlations between resting state EEG gamma band power and scores for depression and depression subtypes based on responses to the Zung Self-rating Depression Scale in a group of 100 depressed and not-depressed participants. Several findings emerged: (1) significant positive correlations between gamma band power and depressive subtypes were only found in the anhedonic depression subtype in the eyes open resting-state condition; (2) in this subtype, voxels that exceeded the significance threshold were predominantly in the left anterior cingulate cortex. Likewise, for overall depression and subsequent subtypes of depression, eyes open resting-state gamma band power was consistently (but not always significantly) most associated with the anterior cingulate cortex; (3) other areas which exceeded the significance threshold between gamma power and anhedonic depression included the posterior cingulate cortex and left middle and superior frontal gyrus; and (4) gamma band power did not correlate with overall depression score.

### 4.1. Anhedonic Depression Subtype and Gamma Band Power

This study revealed significant positive correlations between gamma band power and the anhedonic depression subtype, a subtype marked by withdrawal from reward-seeking behaviours, negative bias, and hypersensitivity to punishment [22]. Anhedonia is a primary symptom of major depressive disorder and is characterised by behavioural deficits in reward processing [7,37,38]. The neural underpinnings of anhedonia are linked to atypical brain function and structure [39,40], including abnormalities in reward circuitry [41,42,43] and the default mode network [44].

While there are no clinical studies comparing gamma activity and the anhedonic depressive subtype, previous findings have indicated links between gamma band activity and aberrant reward processing. There is an established link between gamma band activity and attentional and reward processing in task-based research paradigms [45]. For example, gamma band activity increased after reward delivery during a gambling task [46] and was more pronounced for win-related large rewards compared to small rewards [47]. Gamma activity increase was also observed after unexpected gains, suggesting its involvement in unanticipated positive events [48]. Since there is an absence of rewarding stimuli during resting-state, high gamma band power, as observed in the anhedonic depressive subtype, may be indicative of reward dysfunction in this study.

The current findings suggest that high resting-state gamma activity in anhedonia may result from dysfunctional reward systems. This may also account for the absence of significant correlations between gamma band power and the other three subtypes of depressed mood, cognitive depression, and somatic depression. These subtypes do not have the same level of association with reward behaviours and, therefore, perhaps not the same degree of dysfunction in reward circuitry as the anhedonic subtype [22,49]. While this evidence is preliminary, further investigation in a larger study sample of the association between the gamma band and depressive subtypes could clarify this issue.

### 4.2. Anterior Cingulate Cortex, Depression, Anhedonia, and Gamma Band Power

Results from this study demonstrated that the brain area of most relevance to eyes-open resting-state gamma power and depression was the anterior cingulate cortex (ACC). Very few EEG studies have uncovered significant correlations between gamma band activity and the anterior cingulate cortex. Contrary to the finding of this study, Pizzagalli et al. [50] observed reduced resting state gamma in the anterior cingulate cortex in individuals with elevated depressive symptoms. However, there is a growing body of neuroimaging evidence that the anterior cingulate cortex plays a significant role in MD neuropathology [51,52]. For example, Rodríguez-Cano et al. [53] reported volume reduction in the anterior cingulate cortex and orbitofrontal cortex in major depression. Liu and colleagues [54] found reduced cortical thickness of the left rostral anterior cingulate cortex and lateral orbitofrontal cortex correlated with anhedonia in MD and suggested its use as a biomarker of anhedonia in MD. A review of fMRI studies reported that the anterior cingulate cortex plays a crucial role in depression [55], and Rolls and colleagues [56] found abnormal activation and connectivity between the anterior cingulate cortex and varying brain regions in MD compared to healthy controls.

The anterior cingulate cortex is a crucial brain region for emotional regulation, memory, and reward processes [57]. The rostral anterior cingulate cortex features predominantly in reward circuits of the brain because it receives dense dopaminergic innervation [58] and projects to reward centres such as the striatum, particularly the nucleus accumbens, and ventral tegmental area [59,60]. Findings from Knutson et al. [61], Gorka et al. [62], and Wu et al. [63] confirm that abnormal anterior cingulate cortex activation plays a mediating role in reward dysfunction in depressed patients and is, thus, implicated in anhedonia pathology [64].

Individuals with depression, particularly the anhedonic group, may have a neurocognitive vulnerability toward dysfunctional reward processes and experiencing pleasure, due to atypical activity in the anterior cingulate cortex. The involvement of the anterior cingulate cortex in anhedonia makes it a key region of interest for future studies.

### 4.3. Left Posterior Cingulate and Left Middle and Superior Frontal Gyrus, Depression, and Gamma Band Power

Additional brain areas of significance, which correlated with gamma band power and depressive symptoms in the anhedonia subtype in this study, included the posterior cingulate cortex (PCC) and left middle and superior frontal gyrus, which, along with the anterior cingulate cortex, are regions of the default mode network (DMN) [65,66]. The DMN is a collection of brain regions that are active at rest and deactivated during tasks that demand attention [67,68]. Dai and colleagues [69] observed increased EEG gamma power in regions including the DMN in participants with MD compared to healthy controls. In another EEG study, Pizzagalli, Peccoralo, Davidson, and Cohen [50] reported higher gamma power in the posterior cingulate in individuals with high levels of depression symptoms.

Functional MRI has also highlighted the potential role of the posterior cingulate and DMN in MD. A meta-analysis on functional connectivity reported hyperconnectivity between the PCC and the middle frontal gyrus and hypoconnectivity between the PCC and the superior frontal gyrus in depressed individuals [70]. Additionally, in MD patients with a high level of anhedonia, an fMRI study showed structural and functional differences were apparent in the superior frontal gyrus [39]. Correlations between gamma band power, depression, and regions such as the posterior cingulate cortex and superior frontal gyrus, as seen in the present study, are suggestive of dysfunctional connections and aberrant functioning. Studies involving gamma band power and these additional regions of interest in a larger cohort of subtypes of MD may elucidate this finding.

### 4.4. Null Finding of Overall Depression and Other Subtypes

Previous studies have reported that higher gamma band power correlates with increased total depression [71,72,73,74,75,76]. Contrary to expectation, this was not supported by the data presented here. The failure to find a correlation between gamma and overall depression scores, which contradicts some prior studies [71,72,73,74,75,76], could be a Type II statistical error due to undersampling. For example, as well as a significant correlation between anhedonia subtype and gamma band power, there were small, non-significant, positive correlations between eyes open resting-state gamma band power and overall depression score (*r* = 0.225; *p* = 0.19 two-tailed), and cognitive depression subtype (*r* = 0.219; *p* = 0.208 two-tailed). A larger study with more statistical power may clarify this relationship. It has been suggested that sample sizes of over 200 will yield biologically sound effects and lead to meaningful biomarker discovery [77].

While the current literature related to gamma band activity and depression revealed that high gamma band activity was associated with depression severity, this was not without some discrepancies [16,17]. Both positive and negative correlations between depression and gamma band activity have been reported in recent research [71,78]. The lack of reporting of negative results and publication bias toward positive results may influence these observed inconsistencies [79]. Amongst studies of EEG and depression, the gamma band tends to be the least reported frequency compared to other bands and is also the least clearly defined in terms of bandwidth range [17,80]. Other studies involving gamma band power and depression included a broader range of gamma bands; some studies investigated gamma band up to 200 Hz [16,17], whereas the current study investigated gamma wavelength from 30 to 45 Hz. Thus, analysis of the high gamma band may provide greater insight into the nature of gamma power and depressive subtypes. However, there is no agreed-upon definition of gamma band frequency, and for this study, gamma was restricted to 30–45 Hz due to the greater risk of contamination from facial or cranial muscle activity and environmental noise at higher frequencies [29]. Replication of gamma band studies, including this one, with alternate gamma ranges and/or larger data sets might clarify findings.

### 4.5. Eyes Open and Eyes Closed Gamma Band Power in Depression

This study found significant positive correlations between the eyes open resting-state gamma band power and anhedonic depressive subtype. In the eyes-closed resting-state condition, all correlations between gamma band power and depression and the four subtypes were negative and not significant. Similar to the conclusion drawn by Liu, Liu, Yan, Chen, Liu, Hao, Ou, Huang, Su, He, and Ming [71], the eyes-open resting-state demonstrated more reliable results in gamma band power. It is widely acknowledged that there are differences in arousal states between eyes open and eyes closed resting-states [81]. Petro et al. [82] observed increased gamma band power in the eyes open compared to the eyes closed resting condition, underscoring the distinctiveness of these conditions in the gamma band.

The increase in gamma power with eyes open as opposed to eyes closed may be indicative of heightened rumination in anhedonic patients when exposed to increased sensory information (in this case, visual stimuli). Siegle and colleagues (2010) report depressed patients showed sustained increases in frontal lobe gamma power during and after negative emotion-related word tasks compared to controls [83]. Strelets and colleagues (2007) also report depressed patients displaying increased gamma power in frontal regions during non-emotion-related tasks such as arithmetic and spatial imagination tasks compared to healthy controls [84]. Given gamma’s role in the integration of sensory information [85], these findings indicate people with anhedonic depression may be showing increased gamma responses to visual stimuli (eyes open), whether it is salient or not. Since increased activity in the ACC during rumination-like behaviour has been observed in depressed populations [86,87,88], it is plausible that rumination-like cognition may be increased as a result of increased sensory information in those with anhedonic depression.

## 5. Conclusions

### 5.1. Limitations and Future Directions

Certain limitations of the results should be considered. One limitation includes the cultural identity and locality of the sample. Participants were also volunteers from the community and not part of an identified clinical subgroup. Future studies in different samples and from varied locations will enhance research generalisability. Psychometric findings from previous studies have confirmed that the Zung SDS is a reliable method of measuring depressive symptoms, but the use of clinical interviews or alternative self-report instruments could provide greater reliability [24,26,27]. Diagnostic instruments may, for example, give a more comprehensive picture of depressive subtype symptoms, adding greater validity to the overall results. Additional evaluation of depressive symptoms, mood alterations, and temperament, such as the contribution of anxiety or further depression subtype analysis [89], may have yielded additional value. Anhedonia is often associated with a severe depression profile and treatment resistance, hence further studies will improve the personalised approach to this particular depressive subtype [12,90]. While the sample size met the minimum requirement for correlational analysis, future studies working with a larger sample size should be performed to verify current findings [77]. Indeed, as EEG resting state gamma power has been shown to change with age [91], a larger sample size would provide useful data around age, gamma power, and depression subtype. The study took a “snapshot” approach to depressive symptoms, with data collection occurring at a single moment in time. Sampling of mood states with greater frequency would enhance research generalisability. Longitudinal resting-state assessment of depressive symptoms and the gamma band may further describe disease progression, and the physiological and pathological mechanisms of gamma band activity in anhedonic depression and MD. A longitudinal approach may show whether gamma power abnormalities are trait or state dependent in depression and its subtypes [92]. EEG is an excellent measure of neurocognitive activity with exceptional reliability [93], but this could be complemented through alternative investigative measures (e.g., fMRI). Additionally, an increased number of EEG electrodes may increase the accuracy of brain region localisation and improve results. The method of subtype identification used in this study was a priori since the subtypes were grouped by common depressive symptomologies identified by a panel of experienced clinicians [22]. Identifying subtypes a posteriori by applying cluster analysis then using regression analysis of that data, via a depression scale like the SDS, is another method [94]. Both methods are valuable for defining depressive subtypes, and neither method has been found superior.

Anhedonia can exist apart from depression in conditions including schizophrenia, post-traumatic stress disorder, and eating disorders [95] and in patients in remission from MD [96,97]. For this reason, gamma activity and anhedonia could also be investigated in populations apart from MD as a way of identifying similarities and differences between conditions. These extensions of the research presented here could develop a more comprehensive model of the associations between gamma activity and depression.

### 5.2. Clinical Implications and Conclusions

Depression is one of the foremost mental health issues of the coming decade [1,2], and the heterogeneity of depressive symptoms hinders treatment outcomes in patients [20]. Nonetheless, research is promoting a greater understanding of depression, at least in part, through advances in neuroimaging [97]. The research conducted here used correlational analysis between EEG gamma band power and depression scores on the SDS. Although gamma band power did not show a significant correlation with total depression score or the depressive subtypes, depressed mood, cognitive and somatic depression, it significantly correlated with anhedonic symptoms and identified distinct regions of interest, specifically the left anterior cingulate cortex. These preliminary associations require further validation in larger sample sizes and in clinically characterised groups. The current study is another demonstration that groupings of MD symptoms are likely to be more beneficial than a single inventory score or clinical interview, which assumes a unitary construct of depression. It supports the move from depression as a one-size-fits-all diagnosis and treatment, toward a more personalised or precision approach.

## Figures and Tables

**Table 1:** Four depression subtype profiles and relevant Zung SDS items validated by Sharpley and Bitsika [23].

Subtype	Depressed Mood	Anhedonia	Cognitive	Somatic
Profile	Worthlessness, withdrawal from adverse environments	Withdrawal from reward-seeking, negative bias	Executive functioning deficits, negative emotions	Disrupted appetite, metabolism, sleep, and energy levels
SDS items	1. I feel downhearted and blue 3. I have crying spells or feel like it 14. I feel hopeful about the future 15. I am more irritable than usual 17. I feel that I am useful and needed 19. I feel that others would be better off if I were dead	5. I eat as much as I used to 6. I still enjoy sex 18. My life is pretty full 20. I still enjoy doing the things I used to	11. My mind is as clear as it used to be 12. I find it easy to do the things I used to do 16. I find it easy to make decisions	4. I have trouble sleeping at night 7. I notice that I am losing weight 8. I have trouble with constipation 9. My heart beats faster than usual 10. I get tired for no reason 13. I am restless and can’t keep still

**Table 2:** Demographic information of participants.

	n	Age (Mean)	SD	Range (Years)	Total SDS (Mean)	SD	Range (Total Score)
Total	100	32.53	14.125	18–75	36.7	11.256	21–66
Male	44	33.57	13.961	18–68	36.18	10.31	23–61
Female	56	31.71	14.326	18–75	37.11	12.024	21–66

**Table 3:** Pearson correlations for age, sex, total SDS and four depressive subtype scores.

	Sex	Total SDS	Mood	Anhedonia	Cognitive	Somatic
Age	−0.065	0.055	0.077	0.104	0.136	−0.015
Sex		0.041	0.059	0.012	−0.007	0.054
Total			0.921 **	0.846 **	0.925 **	0.874 **
Mood				0.678 **	0.831 **	0.748 **
Anhedonia					0.789 **	0.626 **
Cognitive						0.736 **

** Correlation is significant at the 0.01 level (2-tailed).

**Table 4:** Mean scores for depressive subtypes.

Subtype	Depressed Mood	Anhedonia	Cognitive	Somatic
Questions	1, 3, 14, 15, 17, 19	5, 6, 18, 20	11, 12, 16	4, 7, 8, 9, 10, 13
Mean	10.42	7.4	6.55	9.71
SD	3.92	2.71	2.43	3.07

**Table 5:** Pearson’s *r-* and *p*-values in gamma band eyes open resting-state for overall SDS and subtypes scores (n = 100).

	Max *r*-Value	Two-Tailed *p*-Value	Brain Structure	Brodmann Area
Total SDS	0.225	0.19	Anterior Cingulate	24
Depressed Mood	0.175	0.44	Anterior Cingulate	24
Anhedonia	0.298	0.032	Anterior Cingulate	24
Cognitive	0.219	0.208	Anterior Cingulate	24
Somatic	0.164	0.521	Anterior Cingulate	24

**Table 6:** Pearson’s *r-* and *p*-values in gamma band eyes closed resting-state for overall SDS and subtypes scores (n = 100).

	Max *r*-Value	Two-Tailed *p*-Value	Brain Structure	Brodmann Area
Total SDS	0.089	0.516	Middle Frontal Gyrus	6
Depressed Mood	0.106	0.415	Inferior Frontal Gyrus	9
Anhedonia	0.090	0.83	Precentral Gyrus	4
Cognitive	0.128	0.872	Inferior Frontal Gyrus	9
Somatic	0.137	0.414	Middle Frontal Gyrus	6

## Data Availability

The raw data supporting the conclusions of this article will be made available by the authors upon request.

## Abbreviations

The following abbreviations are used in this manuscript:

EEG	Electroencephalogram
MD	Major Depression
DMN	Default mode network
SDS	Zung Self-rating Depression Scale
ACC	Anterior cingulate cortex
